# Supplementary material for: HIV knowledge and access to testing for people with and without disabilities in low‐ and middle‐income countries: evidence from 37 Multiple Indicator Cluster Surveys
Source: J Int AIDS Soc. 2024 Apr 2;27(4):e26239. doi: 10.1002/jia2.26239 (PMC10988115; doi:10.1002/jia2.26239)
Supplement: Supplementary file 1 — Supporting Information [file JIA2-27-e26239-s001.docx]

| HIV/AIDS HA | | |
| --- | --- | --- |
| **HA1**. Now I would like to talk with you about something else.  Have you ever heard of HIV or AIDS? | Yes 1  No 2 | 2*⇨End* |
| **HA2**. HIV is the virus that can lead to AIDS.  Can people reduce their chance of getting HIV by having just one uninfected sex partner who has no other sex partners? | Yes 1  No 2  DK 8 |  |
| **HA3**. Can people get HIV from mosquito bites? | Yes 1  No 2  DK 8 |  |
| **HA4**. Can people reduce their chance of getting HIV by using a condom every time they have sex? | Yes 1  No 2  DK 8 |  |
| **HA5**. Can people get HIV by sharing food with a person who has HIV? | Yes 1  No 2  DK 8 |  |
| **HA6**. Can people get HIV because of witchcraft or other supernatural means? | Yes 1  No 2  DK 8 |  |
| **HA7**. Is it possible for a healthy-looking person to have HIV? | Yes 1  No 2  DK 8 |  |
| **HA8**. Can HIV be transmitted from a mother to her baby:  [A] During pregnancy?  [B] During delivery?  [C] By breastfeeding? | Yes No DK  During pregnancy 1 2 8  During delivery 1 2 8  By breastfeeding 1 2 8 |  |
|  |  |  |
| **HA9**. Check HA8[A], [B] and [C]: At least one ‘Yes’ recorded? | Yes 1  No 2 | 2*⇨HA11* |
| **HA10**. Are there any special drugs that a doctor or a nurse can give to a woman infected with HIV to reduce the risk of transmission to the baby? | Yes 1  No 2  DK 8 |  |
| **HA11**. Check CM17: Was there a live birth in the last 2 years?  Copy name of last birth listed in the birth history (CM18) to here and use where indicated:  Name | Yes, CM17=1 1  No, CM17=0 OR BLANK 2 | 2*⇨HA24* |
| **HA12**. Check MN2: Was antenatal care received? | Yes, MN2=1 1  No, MN2=2 2 | 2*⇨HA17* |
| **HA13**. During any of the antenatal visits for your pregnancy with (***name***), were you given any information about:  [A] Babies getting HIV from their mother?  [B] Things that you can do to prevent getting HIV?  [C] Getting tested for HIV?  Were you:  [D] Offered a test for HIV? | Yes No DK  HIV from mother 1 2 8  Things to do 1 2 8  Tested for HIV 1 2 8  Offered a test for hiv 1 2 8 |  |
| **HA14**. I don’t want to know the results, but were you tested for HIV as part of your antenatal care? | Yes 1  No 2  DK 8 | 2*⇨HA17*  8*⇨HA17* |
| **HA15**. I don’t want to know the results, but did you get the results of the test? | Yes 1  No 2  DK 8 | 2*⇨HA17*  8*⇨HA17* |
| **HA16**. After you received the result, were you given any health information or counselling related to HIV? | Yes 1  No 2  DK 8 |  |
| **HA17**. Check MN20: Was the child delivered in a health facility? | Yes, MN20=21-36 OR 76 1  No, MN20=11-12 or 96 2 | 2*⇨HA21* |
| **HA18**. Between the time you went for delivery but before the baby was born were you offered an HIV test? | Yes 1  No 2 |  |
| **HA19**. I don’t want to know the results, but were you tested for HIV at that time? | Yes 1  No 2 | 2*⇨HA21* |
| **HA20**. I don’t want to know the results, but did you get the results of the test? | Yes 1  No 2 | 1*⇨HA22*  2*⇨HA22* |
| **HA21**. Check HA14: Was the respondent tested for HIV as part of antenatal care? | Yes, HA14=1 1  No or no answer, HA14≠1 2 | 2*⇨HA24* |
| **HA22**. Have you been tested for HIV since that time you were tested during your pregnancy? | Yes 1  No 2 | 1*⇨HA25* |
| **HA23**. How many months ago was your most recent HIV test? | Less than 12 months ago 1  12-23 months ago 2  2 or more years ago 3 | 1*⇨HA28*  2⇨*HA28*  3⇨*HA28* |
| **HA24**. I don’t want to know the results, but have you ever been tested for HIV? | Yes 1  No 2 | 2*⇨HA27* |
| **HA25**. How many months ago was your most recent HIV test? | Less than 12 months ago 1  12-23 months ago 2  2 or more years ago 3 |  |
| **HA26**. I don’t want to know the results, but did you get the results of the test? | Yes 1  No 2  DK 8 | 1*⇨HA28*  2*⇨HA28*  8*⇨HA28* |
| **HA27**. Do you know of a place where people can go to get an HIV test? | Yes 1  No 2 |  |
| **HA28**. Have you heard of test kits people can use to test themselves for HIV? | Yes 1  No 2 | 2*⇨HA30* |
| **HA29**. Have you ever tested yourself for HIV using a self-test kit? | Yes 1  No 2 |  |
| **HA30**. Would you buy fresh vegetables from a shopkeeper or vendor if you knew that this person had HIV? | Yes 1  No 2  DK / Not sure / Depends 8 |  |
| **HA31**. Do you think children living with HIV should be allowed to attend school with children who do not have HIV? | Yes 1  No 2  DK / Not sure / Depends 8 |  |
| **HA32**. Do you think people hesitate to take an HIV test because they are afraid of how other people will react if the test result is positive for HIV? | Yes 1  No 2  DK / Not sure / Depends 8 |  |
| **HA33**. Do people talk badly about people living with HIV, or who are thought to be living with HIV? | Yes 1  No 2  DK / Not sure / Depends 8 |  |
| **HA34**. Do people living with HIV, or thought to be living with HIV, lose the respect of other people? | Yes 1  No 2  DK / Not sure / Depends 8 |  |
| **HA35**. Do you agree or disagree with the following statement?  I would be ashamed if someone in my family had HIV. | Agree 1  Disagree 2  DK / Not sure / Depends 8 |  |
| **HA36**. Do you fear that you could get HIV if you come into contact with the saliva of a person living with HIV? | Yes 1  No 2  Says she has HIV 7  DK / Not sure / Depends 8 |  |
